# Screening for *BRCA1*, *BRCA2*, *CHEK2, PALB2*, *BRIP1*, *RAD50*, and *CDH1 *mutations in high-risk Finnish *BRCA1/2-*founder mutation-negative breast and/or ovarian cancer individuals

**DOI:** 10.1186/bcr2832

**Published:** 2011-02-28

**Authors:** Kirsi M Kuusisto, Aleksandra Bebel, Mauno Vihinen, Johanna Schleutker, Satu-Leena Sallinen

**Affiliations:** 1 Institute of Biomedical Technology, University of Tampere, Biokatu 8, Tampere, 33520, Finland; 2 Centre for Laboratory Medicine, Tampere University Hospital, Biokatu 4, Tampere, 33520, Finland; 3 Department of Pediatrics, Genetics Outpatient Clinic, Tampere University Hospital, Biokatu 8, Tampere, 33520, Finland

## Abstract

**Introduction:**

Two major high-penetrance breast cancer genes, *BRCA1 *and *BRCA2*, are responsible for approximately 20% of hereditary breast cancer (HBC) cases in Finland. Additionally, rare mutations in several other genes that interact with *BRCA1 *and *BRCA2 *increase the risk of HBC. Still, a majority of HBC cases remain unexplained which is challenging for genetic counseling. We aimed to analyze additional mutations in HBC-associated genes and to define the sensitivity of our current *BRCA1/2 *mutation analysis protocol used in genetic counseling.

**Methods:**

Eighty-two well-characterized, high-risk hereditary breast and/or ovarian cancer (HBOC) *BRCA1*/*2*-founder mutation-negative Finnish individuals, were screened for germline alterations in seven breast cancer susceptibility genes, *BRCA1*, *BRCA2*, *CHEK2*, *PALB2*, *BRIP1*, *RAD50*, and *CDH1*. *BRCA1*/*2 *were analyzed by multiplex ligation-dependent probe amplification (MLPA) and direct sequencing. *CHEK2 *was analyzed by the high resolution melt (HRM) method and *PALB2, RAD50, BRIP1 *and *CDH1 *were analyzed by direct sequencing. Carrier frequencies between 82 (HBOC) *BRCA1*/*2*-founder mutation-negative Finnish individuals and 384 healthy Finnish population controls were compared by using Fisher's exact test. *In silico *prediction for novel missense variants effects was carried out by using Pathogenic-Or-Not -Pipeline (PON-P).

**Results:**

Three previously reported breast cancer-associated variants, *BRCA1 *c.5095C > T, *CHEK2 *c.470T > C, and *CHEK2 *c.1100delC, were observed in eleven (13.4%) individuals. Ten of these individuals (12.2%) had *CHEK2 *variants, c.470T > C and/or c.1100delC. Fourteen novel sequence alterations and nine individuals with more than one non-synonymous variant were identified. One of the novel variants, *BRCA2 *c.72A > T (Leu24Phe) was predicted to be likely pathogenic *in silico*. No large genomic rearrangements were detected in *BRCA1/2 *by multiplex ligation-dependent probe amplification (MLPA).

**Conclusions:**

In this study, mutations in previously known breast cancer susceptibility genes can explain 13.4% of the analyzed high-risk *BRCA1/2*-negative HBOC individuals. *CHEK2 *mutations, c.470T > C and c.1100delC, make a considerable contribution (12.2%) to these high-risk individuals but further segregation analysis is needed to evaluate the clinical significance of these mutations before applying them in clinical use. Additionally, we identified novel variants that warrant additional studies. Our current genetic testing protocol for 28 Finnish *BRCA1/2*-founder mutations and protein truncation test (PTT) of the largest exons is sensitive enough for clinical use as a primary screening tool.

## Introduction

Breast cancer (BrCa) is the most common cancer among women in Finland, with about 4,000 cases diagnosed yearly (Finnish Cancer Registry). It has been estimated that a monogenic trait accounts for 5 to 10% of all BrCa cases [1]. The two major high-penetrance BrCa genes, *BRCA1 *(*breast cancer 1*) and *BRCA2 *(*breast cancer 2*), are responsible for 30% of hereditary breast cancer (HBC) cases worldwide, but only for about 20% in Finland [2-4]. *BRCA2 *mutations have been found to be more common in the Finnish population than *BRCA1 *[5]. In addition to *BRCA1 *and *BRCA2 *mutations, there are certain hereditary cancer syndromes, such as Li-Fraumeni, Cowden, Peutz-Jeghers and diffuse gastric cancer syndromes, associated with a high risk of BrCa [6-9]. However, these syndromes very seldom explain HBC.

*BRCA1 *and *BRCA2 *have many DNA damage response functions in the cell [10]. Therefore, it has been hypothesized that genes coding for proteins that interact with *BRCA1 *or *BRCA2 *or act in the same DNA repair pathway would be likely candidate genes for HBC susceptibility. As expected, *CHEK2 *(*checkpoint kinase 2*), *PALB2 *(*partner and localizer of BRCA2*), *BRIP1 *(*BRCA1-interacting protein 1*), and *RAD50 *(*human homolog of Saccharomyces cerevisiae RAD50*) have been shown to have rare, moderate-risk BrCa-associated variants, which have also been studied in the Finnish population [11-14]. In addition, BrCa-associated variants have been reported in the *CDH1 *(*cadherin-1*) [15].

Although mutations in many genes have been found to predispose an individual to BrCa, approximately 75 to 80% of HBC cases remain unexplained [16]. It is likely that additional BrCa susceptibility gene mutations remain unidentified, especially in the category of moderate- to low-penetrance gene variants that individually confer only minimal risk but that, through multiplicative and/or cumulative effects, can cause relatively high risk for the carriers [17]. Genome-wide association studies (GWAs) have revealed multiple low penetrance, single nucleotide polymorphisms (SNPs) in many genes and chromosomal loci with increased risk of BrCa. For example, SNPs in the *fibroblast growth factor receptor 2 *(*FGFR2*) gene have shown significant association with increased risk among BrCa cases with strong family history [18].

To address the problem of heterogeneous HBC in genetic counseling, we wanted to investigate possible additional mutations in HBC-associated genes. The aim of this study was to screen seven known BrCa susceptibility genes for additional mutations in 82 well-characterized, Finnish, high-risk hereditary breast and/or ovarian cancer (HBOC) individuals tested to be *BRCA1*/*2*-founder mutation negative. In addition, the sensitivity of our current *BRCA1*/*2 *mutation analysis protocol was defined for genetic counseling purposes.

## Materials and methods

### Patients and controls

Index individuals of 82 high-risk Finnish HBOC families were screened for germline alterations in BrCa-associated genes. All individuals had been detected to be founder mutation-negative by minisequencing of the previously known 28 Finnish *BRCA1/2 *mutations and by protein truncation test (PTT) of exon 11 for *BRCA1 *and exons 10 and 11 for *BRCA2*. Study material had been collected from the individuals, who visited the Tampere University Hospital Genetics Outpatient Clinic between January 1997 and May 2008. The hospital district, in the area of Pirkanmaa, consists of over 20% (1.23 million) of the Finnish population. Individuals were chosen to be included in this study according to the following criteria of high-risk HBC: (a) the individual or her first-degree relative (only female family members were included when defining first-degree relatives) had BrCa or ovarian cancer (OvCa) at younger than 30 years of age; or (b) two first-degree relatives in the family had BrCa and/or OvCa and at least one of the cancers had been diagnosed at younger than 40 years of age; or (c) three first-degree relatives in the family had BrCa and/or OvCa and at least one of the cancers had been diagnosed at younger than 50 years of age; or (d) four or more first-degree relatives had BrCa and/or OvCa at any age; or (e) the same individual had BrCa and OvCa. Patient with bilateral BrCa was considered to have two separate cancers. According to these criteria, our study material also included 11 non-affected females in addition to 71 BrCa and/or OvCa patients. We were also able to get blood samples from two affected relatives in 2 out of 11 separate families with healthy index. These relatives with BrCa were screened for the same variant as that identified in the index. The clinical data of the studied individuals are presented in Table 1. As controls, 384 blood samples from anonymous healthy females, collected from the Finnish Red Cross, were used. All individuals have been informed of the analyses, and they have given written consent to use their already existing DNA samples. Permission for the research project has been received from the Ethical Committee of Tampere University Hospital and the National Authority for Medicolegal Affairs.

**Table 1:** Characteristics of the studied individuals

	BrCa	Bil. BrCa	OvCa	BrCa and OvCa	Non-affected
Number of index individuals (n = 82)	57	8	1	5	11
**Age at diagnosis**					
**(BrCa/OvCa)**	n = 57	n = 16^a^	n = 1	n = 10^b^	-
<30	13	0	0	1	-
<40 years	11	2	0	2	-
<50 years	15	7	0	2	-
≥50 years	18	7	1	5	-
**Type of BrCa**	n = 57	n = 16^a^	-	n = 5	-
Ductal	41	8	-	5	-
Intraductal	2	2	-	0	-
Lobular	9	3	-	0	-
Papillary	1	0	-	0	-
Medullary	1	0	-	0	-
Unknown	3	3	-	0	-
**Ductal carcinomas grade known**	n = 38	n = 6		n = 5	-
Grade 1	7	3		0	-
Grade 2	14	3		3	-
Grade 3	17	0		2	-
**ER status known**	n = 51	n = 11	-	n = 4	-
ER +	35	10	-	2	-
ER-	16	1	-	2	-
**PR status known**	n = 50	n = 11	-	n = 4	-
PR+	30	10	-	2	-
PR-	20	1	-	2	-
**HER2, status known**	n = 46	n = 11	-	n = 4	-
HER2+	14	1	-	1	-
HER2-	32	10	-	3	-
**Type of OvCa**	-	-	n = 1	n = 5	-
Serous	-	-	0	0	-
Endometrioid	-	-	0	0	-
Mucinous	-	-	0	2	-
Clear cell	-	-	0	1	-
Other	-	-	0	2	-
Unknown	-	-	1	0	-
**Number of affected (BrCa/OvCa)**					
**first-degree relatives**	n = 57	n = 8	n = 1	n = 5	n = 11
≥2	25	3	1	0	5
≥1	24	4	0	0	6
0	8	1	0	5	0
**Number of affected (BrCa/OvCa)**					
**second-degree relatives**	n = 57	n = 8	n = 1	n = 5	n = 11
≥2	5	0	0	0	4
≥1	11	1	0	0	0
0	41	7	1	5	7

Bil. BrCa, bilateral breast cancer; BrCa, breast cancer; ER, estrogen receptor; HER2, human epidermal growth factor receptor 2; OvCa, ovarian cancer; PR, progesterone receptor. ^a^8 Bilateral breast cancer cases, together 16 cancers, ^b^5 Breast and ovarian cancer cases, together 10 cancers

### Mutation detection

DNA samples of the individuals were kindly received from the Tampere University Hospital Genetics Outpatient Clinic. Mutation screening for *BRCA1*, *BRCA2*, *PALB2*, *BRIP1*, *RAD50*, and *CDH1 *was performed by direct sequencing. Whole-coding regions and exon-intron boundaries were analyzed. Primer sequences for *PALB2*, *BRIP1*, and *RAD50 *have been reported previously [12,13,19]. Primers for *BRCA1 *and *BRCA2 *(excluding previously analyzed exon 11 for *BRCA1 *and exons 10 and 11 for *BRCA2*) and *CDH1 *were designed by using Primer3 software (Rozen and Skaletsky, Whitehead Institute for Biomedical Research, Cambridge, MA, USA) [20]. *CHEK2 *was screened by using high-resolution melt (HRM) analysis on a Bio-Rad platform (Bio-Rad Laboratories Headquarters, Hercules, CA, USA). Sequencing was carried out using the Big Dye Terminator v.3.1 Cycle Sequencing Kit and ABIPRISM 3130 × l Genetic Analyzer (Applied Biosystems, Foster City, CA, USA). Sequences were analyzed with Sequencher v.4.7 software (Gene Codes Corporation, Ann Arbor, MI, USA). Primer sequences, detailed HRM and PCR reaction conditions are available upon request.

Control frequencies were determined for 18 variants by HRM (*CHEK2 *variants), direct sequencing (*BRCA1 *c.4883T > C and *RAD50 *c.1544A > G) and TaqMan^® ^SNP genotyping assays (Applied Biosystems, Foster City, CA, USA) and with an ABI7900 instrument (Applied Biosystems, Foster City, CA, USA). Assays were already designed and functionally tested for the following SNPs: c.8182G > A (rs28897749), c.9976A > T (rs11571833), c.10234A > G (rs1801426), and c.1676A > G (rs152451). As for the c.72A > T, c.814G > A, c.1000T > G, and c.2993G > A (rs45551636) variants, assays were designed by Custom TaqMan^® ^Assay Design Tool (Applied Biosystems, Foster City, CA, USA) according to manufacturer's instructions.

The multiplex ligation-dependent probe amplification (MLPA) analysis was performed for *BRCA1 *and *BRCA2 *(SALSA MLPA kit P002-B1 for *BRCA1 *(lot 0508) and kit P090-A2 for *BRCA2 *(lot 0808), MRC-Holland, Amsterdam, the Netherlands) according to manufacturer's instructions and analyzed with ABIPRISM 3130xl Genetic Analyzer and Genemapper^® ^v.4.0 software (Applied Biosystems, Foster City, CA, USA).

### Statistical analyses

Carrier frequencies between 82 studied individuals and 384 population controls were compared by using Fisher's exact test [21]. All *P*-values were two sided. Odds ratios (OR) were generated by two-by-two table.

### *In silico *prediction of novel missense variants effects

The effects of five novel-coding missense variants, *BRCA2 *c.72A > T (Leu24Phe), *CHEK2 *c.1363G > A (Val455Ile), *PALB2 *c.814G > A (Glu272Lys), *PALB2 *c.1000T > G (Tyr334Asp), and *RAD50 *c.1544A > G (Asp515Gly), were predicted with a number of tools by using Pathogenic-Or-Not-Pipeline (PON-P) [22]. The predictions included those for amino acid tolerance (programs PolyPhen version 2, Sift, PhD-SNP, SNAP) and protein stability (I-Mutant version 3). PON-P allows simultaneous submission of a number of variations and proteins to selected predictors. PON-P utilizes machine learning to combine results from several individual predictions.

### MicroRNA database and BLAST search for novel variants

MicroRNA (miRNA) target site search was performed for the novel variant genomic positions from the microRNA database (miRBase) [23]. Also BLAST search [24] was performed for the novel human variant genomic positions to see if these sites are conserved among different organisms including mouse, rat, cow, and chicken.

## Results

Index individuals of 82 high-risk HBOC families were screened for germline alterations in *BRCA1*, *BRCA2*, *CHEK2*, *PALB2, BRIP1*, *RAD50*, and *CDH1 *genes. Detailed clinical information of analyzed individuals is shown in Table 1. All of the identified 54 sequence variants with their observed genotype frequencies and rs-numbers are presented in Supplementary Table S1 in Additional file 1. All of the identified non-synonymous and novel sequence alterations are summarized in Table 2. Table 2 variants are presented in Table 3 with index individual and family cancer history. In addition, as our study material also included healthy index individuals from 11 families, we made an effort to get blood samples from two affected relatives in 2 out of 11 separate families. These relatives with BrCa were screened for the same variant as that identified in the healthy index. Analysis was performed for the new cases in family 112 (*CHEK2 *c.470T > C and *PALB2 *c.1676A > G variants) and family 231 (*BRCA1 *c.4883T > C variant; Table 3). In family 112, the case proved to have the same *PALB2 *c.1676A > G variant as the index individual but in family 231, the affected relative did not carry the *BRCA1 *c.4883T > C variant (data not shown). To further evaluate the impact of these 11 healthy index cases, we recalculated the frequencies without these 11 individuals for those variants accepted to be meaningful for BrCa risk. Supplementary Table S2 in Additional file 2 shows these re-calculated frequencies for *BRCA1 *c.5095C > T, *CHEK2 *c.470T > C, and *CHEK2 *c.1100delC variants. No statistically significant effect was seen for exclusion of the 11 cases.

**Table 2:** Identified non-synonymous and novel sequence alterations

			Carrier frequency				
						
**Gene/Nucleotide change**^a^	Effect on protein	**rs Number**^b^	Individuals	Controls	*P*-values	OR; 95%CI	Status
***BRCA1***							
4837A > G	Ser1613Gly	rs1799966	0.634 (52/82)^g^	na	-	-	Reported^c, d^
4883T > C	Met1628Thr	rs4986854	0.049 (4/82)	0.016 (6/367)	0.090	3.09; 0.85-11.19	Reported^c, d^
5095C > T	Arg1699Trp	rs55770810	0.012 (1/82)	na	-	-	Reported^c, d^
***BRCA2***							
68-80insT^f^	-	-	0.012 (1/82)	na	-	-	Novel
72A > T	Leu24Phe	-	0.012 (1/82)	0 (0/380)	0.177	na	Novel
793 + 34T > G	-	-	0.012 (1/82)	na	-	-	Novel
8182G > A	Val2728Ile	rs28897749	0.012 (1/82)	0.003 (1/378)	0.325	4.65; 0.29-75.19	Reported^c, d^
9976A > T	Lys3326Stop	rs11571833	0.012 (1/82)	0.029 (11/378)	0.702	0.41; 0.05-3.24	Reported^c, d^
10234A > G	Ile3412Val	rs1801426	0.012 (1/82)	0.021 (8/379)	1.000	0.57; 0.07-4.64	Reported^c, d^
***CHEK2***							
444 + 85T > A	-	-	0.012 (1/82)	0.005 (2/364)	0.457	2.23; 0.20-24.94	Novel
470T > C	Ile157Thr	-	0.098 (8/81)	0.055 (21/381)	0.203	1.88; 0.80-4.41	Reported^e^
792 + 39C > T	-	-	0.012 (1/82)	0.021 (8/375)	1.000	0.57; 0.07-4.60	Novel
1100delC^f^	Fs, stop at codon 381	-	0.037 (3/82)	0.016 (6/380)	0.203	2.37; 0.58-9.67	Reported^e^
1290T > C	His430His	-	0.951 (77/81)^g^	0.974 (372/382)	0.281	0.52; 0.16-1.69	Novel
1314T > C	Asp438Asp	-	0.951 (77/81)^g^	0.974 (372/382)	0.281	0.52; 0.16-1.69	Novel
1363G > A	Val455Ile	-	0.975 (79/81)^g^	0.976 (373/382)	1.000	0.95; 0.20-4.50	Novel
***PALB2***							
814G > A	Glu272Lys	-	0.012 (1/82)	0 (0/372)	0.181	na	Novel
1000T > G	Tyr334Asp	-	0.012 (1/82)	0.011 (4/380)	1.000	1.16; 0.13-10.52	Novel
1010T > C	Leu337Ser	rs45494092	0.073 (6/82)	na	-	-	Reported^c^
1676A > G	Gln559Arg	rs152451	0.122 (10/82)	0.173 (64/371)	0.323	0.67; 0.33-1.36	Reported^c^
2205A > G	Pro735Pro	-	0.012 (1/82)	na	-	-	Novel
2794G > A	Val932Met	rs45624036	0.037 (3/82)	na	-	-	Reported^c^
2993G > A	Gly998Glu	rs45551636	0.012 (1/82)	0.038 (14/372)	0.491	0.32; 0.04-2.44	Reported^c^
***BRIP1***							
584T > C	Leu195Pro	rs4988347	0.024 (2/82)	na	-	-	Reported^c^
2755C > T	Pro919Ser	rs4986764	0.390 (32/82)^g^	na	-	-	Reported^c^
***RAD50***							
1544A > G	Asp515Gly	-	0.012 (1/82)	0.010 (4/384)	1.000	1.17; 0.13-10.63	Novel
2398-32A > G	-	-	0.012 (1/82)	na	-	-	Novel
3475 + 33C > G	-	-	0.012 (1/82)	na	-	-	Novel

CI, confidence interval; Fs, frameshift; na, not analyzed; OR, odds ratio. ^a^The reference nucleotide sequencies were obtained from the UCSC Genome Browser [44] and the accession numbers were following: *BRCA1*: [UCSC Genome Browser:NM_007295.2], *BRCA2*: [UCSC Genome Browser:NM_000059.3], *CHEK2*: [UCSC Genome Browser:NM_007194.3], *PALB2*: [UCSC Genome Browser:NM_024675.3], *BRIP1*: [UCSC Genome Browser:NM_032043.1], *RAD50*: [UCSC Genome Browser:NM_005732.3], and *CDH1*: [UCSC Genome Browser:NM_004360.3]. The accession numbers for the protein sequencies obtained from the Swiss-Prot Protein knowledgebase [45] were following: *BRCA1*: [Swiss-Prot:P38398], *BRCA2*: [Swiss-Prot:P51587], *CHEK2*: [Swiss-Prot:O96017], *PALB2*: [Swiss-Prot:Q86YC2], *BRIP1*: [Swiss-Prot:Q9BX63], *RAD50*: [Swiss-Prot:Q92878], and *CDH1*: [Swiss-Prot:P12830]. ^b^The RefSNP number, obtained from the NCBI Single Nucleotide Polymorphism database (dbSNP) [46]. ^c^The NCBI dbSNP [46]. ^d^The Breast Cancer Information Core database [47]. ^e^Reported in the Finnish population by Vahteristo et al. [11]. ^f^Heterozygous deletion or insertion.^g^Due to the high frequency of the variant observed in analyzed individuals, variant is not presented in Table 3.

**Table 3:** Identified variants in the studied individuals

Family id	Gene and variant	Type of cancer	BrCa/OvCa Histology,Grade	Receptor status	Other cancer cases in the family (Age at diagnosis if available)
202	*BRCA1*, 4883T > C	Br (26)	Ductal, 3	ER-, PR-, HER2-	Skin (54)
206	*BRCA1*, 4883T > C	Br (53)	Ductal, 1	ER-, PR-, HER2-	Bil. Ov (64), Br (49)
231	*BRCA1*, 4883T > C	-			Br x2 (33, 46), Cer (60), Skin (73)
232	*BRCA1*, 4883T > C	Br (34)	Ductal, na	ER +, PR +, HER2 na	Br (39)
249 (Figure 1)	*BRCA1*, 5095C > T	Br (42)	Medullary, na	na	Br x5 (*35*, **44**, 57, 67, 71, ),
					Co (78), Kid (67), Mel (63), *Ov (45)*,
					Skin, To (51), Ute (39)
115	*BRCA2*, 68-80insT	Br (59)	Lobular, 2	ER+, PR+, HER2-	Br x3 (<50), Br x2 (60, 60)
240	*BRCA2*, 72A > T	Br (53)	Ductal, 3	ER+, PR-, HER2+	Br x2 (42, 62)
207	*BRCA2*, 793 + 34T > G	Br (38)	na	na	Bil. Br (64)
5 (Figure 8)	*BRCA2*, 8182G > A	-			Bil. Br x2 (43, 48 and 54, 76),
	*BRCA2*, 10234A > G				Br (43), *Brain (75)*, Lip (45), *Lung (81)*, *Skin (75)*, Sto (56)
4	*BRCA2*, 9976A > T	-			Bil. Br x2 (53, 69 and <70),
	*CHEK2*, 470T > C				Br (<70)
212	*CHEK2*, 444 + 85T > A	Bil. Br (43)	Ductal, 2 and na	ER+, PR+, HER2- and	Br (52)
	*PALB2*, 2794G > A			na	
110 (Figure 3)	*CHEK2*, 792 + 39C > T	Br (26)	Ductal, 2	ER+, PR+, HER2+	*Br (48)*, Ca (84), *Pr (64)*
	*CHEK2*, 470T > C				
	*CHEK2*, 1100delC				
	*RAD50*, 2398-32A > G				
112	*CHEK2*, 470T > C	-			Br x3 (35, 43, 83), Skin (76),
	*PALB2*, 1676A > G				*Lung (71)*
120	*CHEK2*, 470T > C	-			Br (64), Ov (72)
122	*CHEK2*, 470T > C	Br (25)	Ductal, 2	ER-, PR-, HER2+	*Brain (66*, *Ca (83)*,C*er (31)*,
					Pr (93), Re (73), Skin (87)
126	*CHEK2*, 470T > C	Br (48)	Ductal, na	ER, PR, HER2 na	*Bil. Br*, Br x2 (51, <*53*)
129 (Figure 2)	*CHEK2*, 470T > C	Skin (70),	Lobular, 2 and	ER-, PR-, HER2- and	Bil. Br (59), **Co (58)**, **Skin (48)**
	*PALB2*, 1676A > G	Bil. Br (78),	Ductal, 1	ER+, PR+, HER2-	
		Sto (82)			
262 (Figure 6)	*CHEK2*, 470T > C	Bil. Br	Intraductal, na and	ER+, PR+, HER2+ and	Br (57), Panc (83), *Si (79)*
	*PALB2*, 1000T > G	(45, 58)	Ductal, na	ER+, PR+, HER2-	
264 (Figure 4)	*CHEK2*, 1100delC	Bil. Br (44)	Lobular, 2	ER+, PR+, HER2-	Br x2 (44, *52*)
265 (Figure 5)	*CHEK2*, 1100delC	Br (45)	Ductal, 3	ER+, PR+, HER2+	Br (38)
	*PALB2*, 1676A > G				
237	*PALB2*, 814G > A	Br (28)	Ductal, 2	ER+, PR+, HER2+	-
133	*PALB2*, 1010T > C	Br (48)	Ductal, 2	ER+, PR+, HER2-	*Int, Br x2 (73, 79)*, Skin (60)
235	*PALB2*, 1010T > C	Br (52)	na	na	Bil. Br (28), Br (56)
239	*PALB2*, 1010T > C	Br (37)	Ductal, 2	ER+, PR+, HER2-	*Br ( > 90)*
250	*PALB2*, 1010T > C	Br (24)	Ductal, 3	ER+, PR+, HER2+	Cer (30), *Ov (83)*
260	*PALB2*, 1010T > C	Br (29)	Ductal, 3	ER-, PR-, HER2-	Br (58), *Lung (60)*
267	*PALB2*, 1010T > C	Br (48)	Ductal, 1	ER+, PR+, HER2 na	Br x2 (51, 58)
113	*PALB2*, 1676A > G^a^	Br (51),	Ductal, 3	ER-, PR-, HER2+	Br (35)
		Skin (55)			
131 (Figure 7)	*PALB2*, 1676A > G	Bil. Br (54)	Intraductal, na and	na	Bil. Br (46), Br (48)
	*BRIP1*, 584T > C		Ductal, 2		
229	*PALB2*, 1676A > G	Bil. Br (68)	na	na	Bil. Br (50, 70), Br x2 (45, 50)
236	*PALB2*, 1676A > G	Br (29)	Intraductal, na	na	Br (52)
246	*PALB2*, 1676A > G	Thy (30),	Ductal, 3	ER-, PR-, HER2+	Br x2 (49, 54), *Re (61)*
		Cer (33),			
		Br (39)			
268	*PALB2*, 1676A > G	Br (62)	Papillary, na	ER+, PR+, HER2-	Br x2 (36, 38)
	*PALB2*, 2205A > G				
271	*PALB2*, 1676A > G	Thy (62),	Lobular, 2	ER+, PR+, HER2-	**Br x2 (43, 44)**
		Br (65)			
102	*PALB2*, 2794G > A	Br (29)	Lobular, na	ER-, PR-, HER2+	*Br (72)*
	*BRIP1*, 584T > C				
244	*PALB2*, 2794G > A	Br (45)	Ductal, 2	ER+, PR+, HER2-	Bil. Br (<45), Br x2 (<35, 46),
					Brain (67)
270	*PALB2*, 2993G > A	Br (66)	Ductal, 3	ER+, PR+, HER2-	Br x2 (48, *<66*)
257	*RAD50*, 1544A > G	Br (39)	Lobular, 2	ER+, PR+, HER2-	Br (69)
225	*RAD50*, 3475+33C > G	Br (43)	Ductal, 1	ER+, PR+, HER2-	Br x2 (52, 77), *Kid (64)*

ER, estrogen receptor; PR, progesterone receptor; HER2, human epidermal growth factor receptor 2; na, not available; Bil, Bilateral; Br, breast; Ca, cancer with unknown primary site; Cer, cervix; Co, colon; Int, intestines; Kid, kidney; Mel, melanoma; Ov, ovary; Panc, pancreas; Pr, prostate; Re, rectum; Si, Sigma; Sto, stomach; Thy, thyroid; To, tongue; Ute, uterus. ^a^Homozygous variant. *Cancers diagnosed in the paternal side of the family are presented in italics*. Cancers diagnosed in siblings or their children of the index patients are underlined. **Cancers diagnosed in the children of the index patients are presented in bold**.

### *BRCA1 *and *BRCA2 *mutation analysis

**Figure 1:**
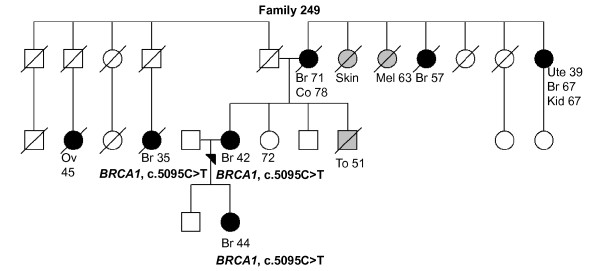
**Family 249 pedigree**. Family pedigree of the index individual with the identified *BRCA1 *c.5095C > T variant (same variant was also identified in the daughter of the index individual and in the daughter of the index individual's paternal uncle). Individuals with breast or ovarian cancer with age at diagnosis are marked with black circles. Other cancers are marked in grey and accompanied by age at diagnosis, if known. Index individual is marked with an arrow. Deceased individuals are indicated with a slash. Current ages of healthy females are marked if known. Br, breast cancer; Co, colon; Kid, kidney; Mel, melanoma; Ov, ovarian cancer; To, tongue; Ute, uterus.

Analysis of *BRCA1 *and *BRCA2 *revealed altogether 16 different sequence variants, seven in *BRCA1 *and nine in *BRCA2 *[see Supplementary Table S1 in Additional file 1]. All but two of the identified variants in *BRCA1*, c.4883T > C and c.5095C > T, have been reported to be neutral in the databases. Heterozygous c.4883T > C variant was observed in 4 of 82 (4.9%) women of which three had BrCa and one had a family history of breast, cervix and skin cancers (Tables 2 and 3). In population controls, the frequency of the c.4883T > C variant was 6 of 367 (1.6%). The c.5095C > T variant has been classified as a deleterious mutation in the Breast Cancer Information Core (BIC) database. The heterozygous c.5095C > T mutation was observed in 1 of 82 (1.2%) women. The mutation carrying woman had BrCa diagnosed at the age of 42 years and a strong family history of cancer (Tables 2 and 3, Figure 1, Family 249). Additional mutation analysis also revealed two other affected women carrying the c.5095C > T mutation in the same family. In *BRCA2*, three of the nine identified variants were novel, c.68-80insT, c.72A > T, and c.793 + 34T > G (Tables 2 and 3.). The heterozygous missense variant c.72A > T (Leu24Phe), was observed in 1 of 82 (1.2%) women but not in population controls. The c.72A > T variant carrying woman had BrCa diagnosed at the age of 53 years. She had also two affected first-degree relatives (mother and sister). Protein predictions by PON-P suggested that substitution of leucine by phenylalanine in position 24 changes significantly the properties of the side chain and the substitution would not be tolerated. All the other identified variants in *BRCA2 *have been reported previously and they are either neutral or the clinical significance of the variants is yet uncertain especially with the three missense variants, c.8182G > A, c.9976A > T and c.10234A > G (Tables 2 and 3). No deletions or duplication were identified either in *BRCA1 *or *BRCA2 *by multiplex ligation-dependent probe amplification (MLPA).

### *CHEK2 *mutation analysis

**Figure 2:**
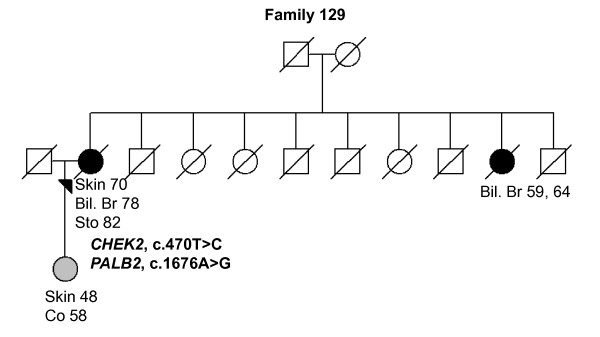
**Family 129 pedigree**. Family pedigree of the index individual with the identified *CHEK2 *c.470T > C and *PALB2 *c.1676A > G variants. Individuals with breast cancer with age at diagnosis are marked with black circles. Other cancers are marked in grey and accompanied by age at diagnosis, if known. Index individual is marked with an arrow. Deceased individuals are indicated with a slash. Bil. Br, bilateral breast cancer; Co, colon; Sto, stomach.

**Figure 3:**
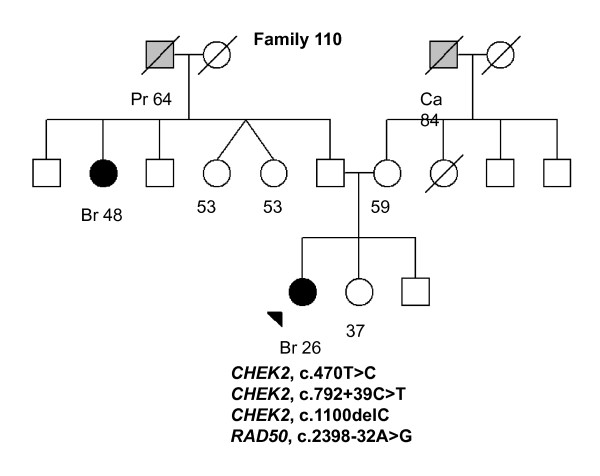
**Family 110 pedigree**. Family pedigree of the index individual with the identified *CHEK2 *c.470T > C, c.792 + 39C > T, c.1100delC, and *RAD50 *c. 2398-32A > G variants. Individuals with breast cancer with age at diagnosis are marked with black circles. Other cancers are marked in grey and accompanied by age at diagnosis, if known. Index individual is marked with an arrow. Deceased individuals are indicated with a slash. Current ages of healthy females are marked if known. Br, breast cancer; Ca, cancer with unknown primary site; Pr, prostate.

**Figure 4:**
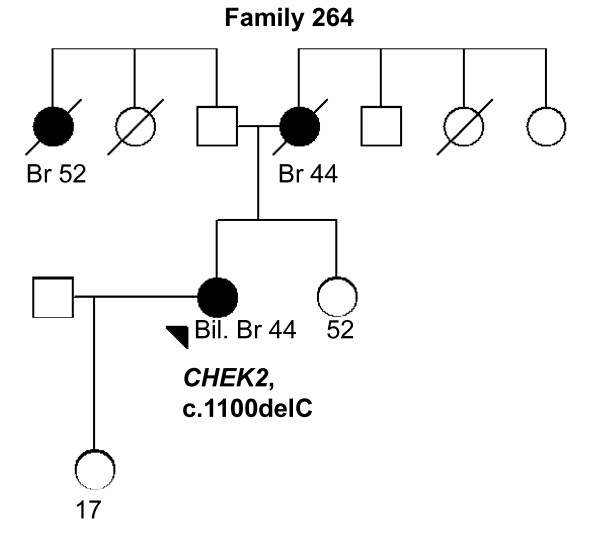
**Family 264 pedigree**. Family pedigree of the index individual with the identified *CHEK2 *c.1100delC variant. Individuals with breast cancer with age at diagnosis are marked with black circles. Index individual is marked with an arrow. Deceased individuals are indicated with a slash. Current ages of healthy females are marked if known. Bil. Br, bilateral breast cancer; Br, breast cancer.

**Figure 5:**
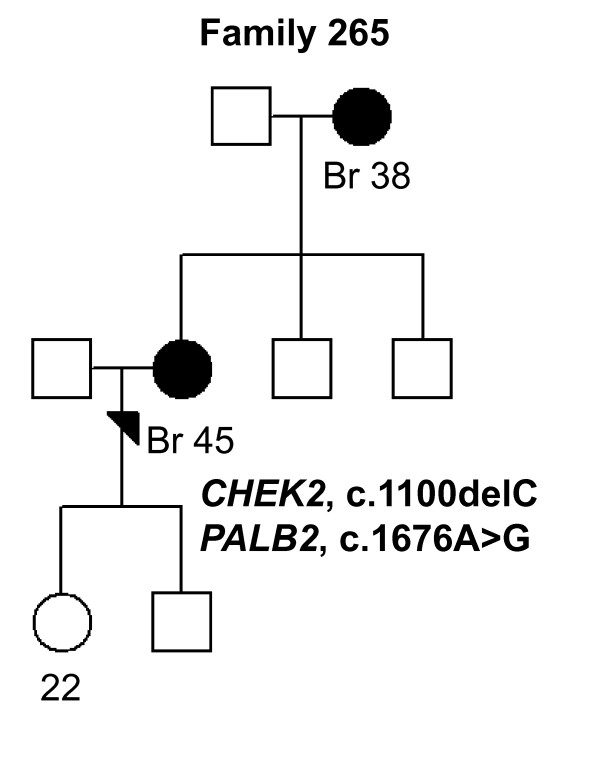
**Family 265 pedigree**. Family pedigree of the index individual with the identified *CHEK2 *c.1100delC and *PALB2 *c.1676A > G variants. Individuals with breast cancer with age at diagnosis are marked with black circles. Index individual is marked with an arrow. Current ages of healthy females are marked if known. Br, breast cancer.

In *CHEK2*, two previously reported BrCa-associated variants in the Finnish population, c.470T > C and c.1100delC, were identified in 10 of 82 (12.1%) individuals (Tables 2 and 3). The heterozygous c.470T > C variant was observed in eight women of which three were healthy. Two of the c.470T > C variant carriers had bilateral BrCa and they carried also *PALB2 *missense variants (an example of the family pedigree of the index individual carrying the both variants is presented in Figure 2, Family 129). The heterozygous c.1100delC variant was detected in three women (Tables 2 and 3). One woman carrying c.1100delC with an early-onset disease of 26 years of age also carried the c.470T > C and the novel c.792 + 39C > T *CHEK2 *variants as well as the *RAD50*, c.2398-32A > G variant (Figure 3, Family 110). A second patient with the c.1100delC variant had bilateral BrCa at the age of 44 years and two other affected individuals in her family (mother and father's sister; Figure 4, Family 264). A third patient with the c.1100delC variant had BrCa diagnosed at the age of 45 years and one affected individual (mother) in her family. This woman carried also the *PALB2 *c.1676A > G variant (Figure 5, Family 265). In addition to c.470T > C and c.1100delC, five novel variants (Table 2) and one common polymorphism [see Supplementary Table S1 in Additional file 1] were identified in *CHEK2*. The novel non-synonymous variant, c.1363G > A (Val455Ile), is based on the computational predictions, and is likely benign.

### *PALB2 *mutation analysis

**Figure 6:**
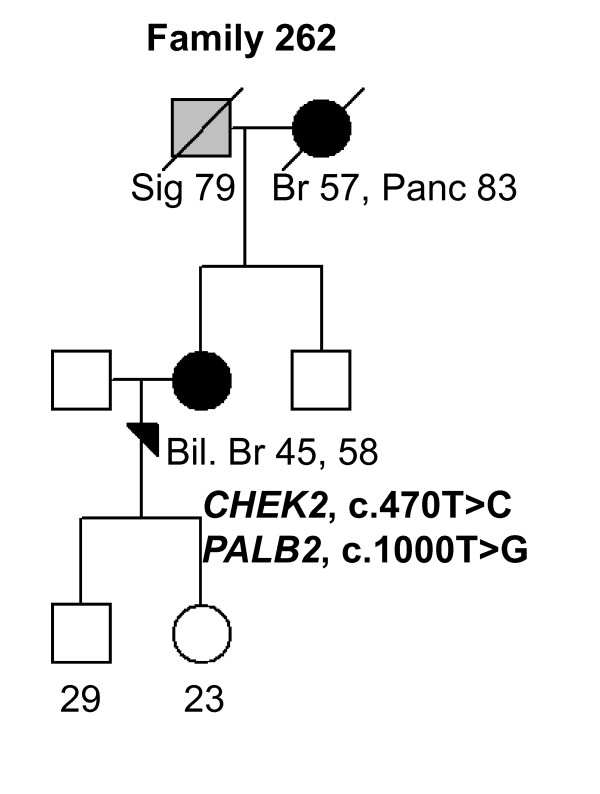
**Family 262 pedigree**. Family pedigree of the index individual with the identified *CHEK2 *c.470T > C and *PALB2 *c.1000T > G variants. Individuals with breast cancer with age at diagnosis are marked with black circles. Other cancers are marked in grey and accompanied by age at diagnosis, if known. Index individual is marked with an arrow. Deceased individuals are indicated with a slash. Current ages of healthy females are marked if known. Bil. Br, bilateral breast cancer; Br, breast cancer, Panc, pancreas; Si, sigma.

**Figure 7:**
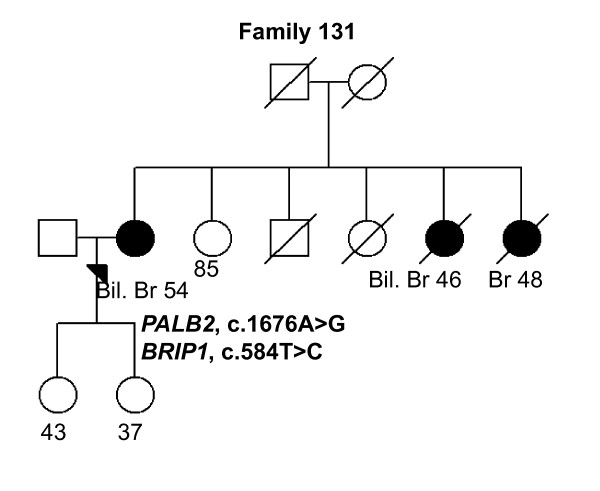
**Family 131 pedigree**. Family pedigree of the index individual with the identified *PALB2 *c.1676A > G and *BRIP1 *c.584T > C variants. Individuals with breast cancer with age at diagnosis are marked with black circles. Index individual is marked with an arrow. Deceased individuals are indicated with a slash. Current ages of healthy females are marked if known. Bil. Br, bilateral breast cancer; Br, breast cancer.

In *PALB2*, altogether nine different variants, including three novel ones, were identified [see Supplementary Table S1 in Additional file 1]. Only one of the identified variants reported previously, c.2586 + 58C > T, has been associated with a 36% increase of BrCa risk (odds ratio (OR): 1.36; 95% confidence intervals (CIs), 1.13-1.64; *P *= 0.001) in a Chinese population [25]. We identified the c.2586 + 58C > T variant in 36 of 82 (43.9%) women. A novel heterozygous c.814G > A variant was identified in 1 of 82 (1.2%) women but not in population controls (Tables 2 and 3). The c.814G > A variant carrying woman had BrCa diagnosed at the age of 28 years, but no other affected individuals were seen in her family. The c.814G > A variant results in amino acid substitution of glutamic acid to lysine at position 272, which causes a significant change to side chain properties including size and change of the charge to opposite. However, protein predictions by PON-P suggest that variation is neutral. The second novel heterozygous variant, c.1000T > G (Tyr334Asp), was observed in 1 of 82 (1.2%) women and in 4 of 380 (1.1%) population controls. The c.1000T > G variant carrying woman had bilateral BrCa diagnosed at the ages of 45 and 58 years and a family history of three other cancers (Tables 2 and 3, Figure 6, Family 262). She carried also the *CHEK2 *c.470T > C variant. However, the protein predictions for the c.1000T > G (Tyr334Asp) variant suggest it to be neutral. A third novel heterozygous variant, c.2205A > G (Pro735Pro), is silent and likely to be neutral. It was observed in 1 of 82 (1.2%) women (Tables 2 and 3). Previously reported *PALB2 *missense variants, c.1010T > C, c.1676A > G, c.2794G > A, and c.2993G > A were identified here with frequencies from 1.2% to 12.2% in analyzed individuals (Tables 2 and 3) but the variants have not been associated with BrCa risk (an example of the family pedigree of the index individual carrying the c.1676A > G variant in addition to the *BRIP1 *c.584T > C variant is presented in Figure 7, Family 131).

### *BRIP1*, *RAD50*, and *CDH1 *mutation analysis

In *BRIP1*, two silent [see Supplementary Table S1 in Additional file 1] and two missense variants (Tables 2 and 3) were identified. All of the identified variants have been reported previously and they are likely to be neutral. In *RAD50*, altogether seven sequence alterations were observed [see Supplementary Table S1 in Additional file 1] and three of these were novel (Table 2). The novel missense variant, c.1544A > G (Asp515Gly), was observed in 1 of 82 (1.2%) women and in 4 of 384 (1.1%) population controls. The c.1544A > G variant carrying woman had BrCa diagnosed at the age of 39 years and one affected first-degree relative (Table 3). According to protein predictions, c.1544A > G variant is likely to be neutral. Two other novel variants, c.2398-32A > G and c.3475 + 33C > G, were both observed with the frequency of 1 of 82 (1.2%) in analyzed individuals (Tables 2 and 3). In *CDH1*, 10 different sequence alterations were identified [see Supplementary Table S1 in Additional file 1]. All of the variants have been reported previously and they are likely neutral.

### MicroRNA database and BLAST search for novel variants

No known miRNA target sites were found in the identified novel variant genomic positions. In BLAST search, *BRCA2 *c.72A > T variant position was found to have sequence similarities between rat and cow. *RAD50 *c.1544A > G variant position shared similarities with mouse, rat, cow and chicken. Three novel variant positions in *CHEK2 *exon 11 and the *RAD50 *c.3475 + 33C > G variant shared sequence similarity between mouse, rat and cow. Variants that occur in the genomic regions that are conserved across species may indicate a pathogenic role.

## Discussion

In the present study, we screened BrCa susceptibility genes in 82 Finnish high-risk HBOC individuals with no known Finnish *BRCA1*/*2-*founder mutations. As genetic counseling and surveillance is greatly needed for these individuals and their families, we decided to study *BRCA1/2 *in more detail and also to analyze five additional genes that had previously been associated with BrCa risk.

**Figure 8:**
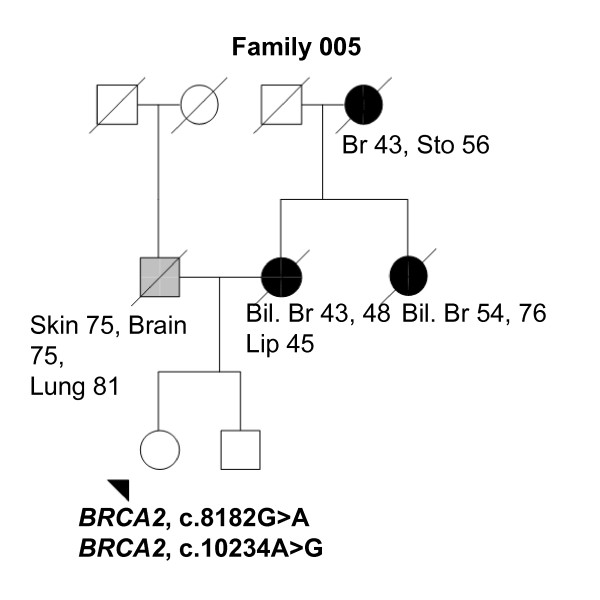
**Family 005 pedigree**. Family pedigree of the index individual with the identified *BRCA2 *c.8182G > A and c.10234A > G variants. Individuals with breast cancer with age at diagnosis are marked with black circles. Other cancers are marked in grey and accompanied by age at diagnosis, if known. Index individual is marked with an arrow. Deceased individuals are indicated with a slash. Bil. Br, bilateral breast cancer; Br, breast cancer; Sto, stomach.

The majority of known *BRCA1/2 *alterations are small insertions and deletions or point mutations (BIC database). Also, large genomic rearrangements have been reported in both genes with varying frequencies in different populations [26]. In Finland, so far only Pylkäs *et al. *have reported a large deletion in *BRCA1 *identified in a Finnish OvCa family [27]. In our study, no deletions or duplications were found in either *BRCA1 *or *BRCA2 *by MLPA, which suggests the existence of more restricted alterations. A total of 16 different sequence variants were identified from these two genes [see Supplementary Table S1 in Additional file 1] and only one of the identified variants, c.5095C > T in *BRCA1*, has been classified as a clinically significant mutation in the BIC database. In line with this classification, our BrCa patient carrying this variant had a strong family history of cancer (Tables 2 and 3, Figure 1, Family 249) and two other variant carriers with BrCa were also observed in the same family. The c.5095C > T mutation thus can explain a fraction of the BrCa cases also in the Finnish population. The clinical significance of the *BRCA1 *c.4883T > C variant in BrCa predisposition is uncertain [28,29]. Our data support the idea that it is a low-penetrant risk allele, because the variant was observed to be three times more common in analyzed high-risk individuals than healthy population controls (Tables 2 and 3). Novel variant findings in *BRCA2 *(Tables 2 and 3) warrant additional studies, especially the novel missense variant, c.72A > T (Leu24Phe), which was shown not to be tolerated by protein prediction. Prediction indicated that the substitution decreases the stability of the produced protein and this might be the mechanism behind the disease for this variant. The amino acid position 24 is located near the N-terminal part of BRCA2. Amino acids 1 to 40 interact with PALB2, and sequence variants in this region have been shown to have effects on the PALB2 and BRCA2 interaction and thus are suspected to have a role in cancer predisposition [30]. The role of the three *BRCA2 *missense variants, c.8182G > A, c.9976A > T, and c.10234A > G, in HBOC risk, is uncertain [31-33]. All three heterozygous variants were observed in two healthy women with a history of BrCa, one carrying the c.9976A > T variant and the other both the c.8182G > A and c.10234A > G variants (Tables 2 and 3, Figure 8, Family 005). At this stage, because we only have samples from the index individuals, no segregation analyses of the variants have been performed, but these families clearly warrant additional studies. In recent risk models, it has been suggested that multiple low-risk variants within the same individual may actually cause a significantly elevated risk for the carrier [17]. The overall low frequency of new variants identified in *BRCA1/2 *genes suggests that the present protocol for testing 28 Finnish *BRCA1/2*-founder mutations and PTT of the largest exons is adequate for clinical use to detect the majority of harmful mutations in these two genes in the Finnish population.

Two of the *CHEK2 *variants, c.470T > C and c.1100delC, have been widely studied in BrCa predisposition in Finland and elsewhere. Previous studies have shown that the c.1100delC allele confers about a two-fold elevated BrCa risk in women, whereas c.470T > C is a lower risk variant [34,35]. Both variants also associate with other cancers in the Finnish population [36-38]. In our study, two of the *CHEK2 *variants, c.470T > C and c.1100delC, were identified in 10 out of 82 analyzed individuals (12.2%) suggesting that the contribution of the two *CHEK2 *variants to BrCa risk is remarkable in the high-risk Finnish *BRCA1/2*-founder mutation-negative individuals. However, clinical screening of the *CHEK2 *variants has not yet been justified due to unclear clinical consequences related to incomplete segregation of the variants with BrCa in the high-risk BrCa families [39,40]. Based on the findings of this study, we agree that interpretation of the *CHEK2 *mutation analysis results is very difficult, because many other gene variants were also identified in individuals with either c.470T > C or c.1100delC variants and some of the variant carriers had not (yet) been diagnosed with BrCa. Thus profound segregation analysis of the c.470T > C and c.1100delC variants for *BRCA1/2*-founder mutation-negative families would be needed to further study clinical significance of these variants. Also the novel variants identified in *CHEK2 *should be further analyzed.

*PALB2 *has been associated with BrCa predisposition in Finland by Erkko *et al. *[12] and the c.1592delT variant was classified as a Finnish founder mutation. In this study the founder deletion was not found, which is probably explained by the limited number of analyzed high-risk HBOC individuals. We identified two novel *PALB2 *missense variants, c.814G > A (Glu272Lys) and c.1000T > G (Tyr334Asp), in affected individuals (Tables 2 and 3). Protein predictions suggested a non-pathogenic role of these substitutions but further studies are needed to confirm these findings. None of the four previously reported *PALB2 *missense variants, c.1010T > C, c.1676A > G, c.2794G > A, and c.2993G > A, have been associated with BrCa risk [12,41]. Interestingly, these variants were identified also together with other variants in analyzed individuals (Tables 2 and 3). One of the identified intronic variants, c.2586 + 58C > T, has been associated with an increase of BrCa risk in a Chinese population [25] but there is no evidence of that in the Finnish population.

*BRIP1 *and *RAD50 *genes have been shown to have rare BrCa associated variants in familial BrCa patients [14,42]. Here, *BRIP1 *mutation analysis revealed only previously reported likely neutral variants. Whereas analysis of *RAD50 *identified three novel sequence alterations, including one missense variant, c.1544A > G (Asp515Gly) (Tables 2 and 3). To further study the role of these novel variants, additional analyses are needed. Germline mutations in *CDH1 *have been previously found to associate with hereditary diffuse gastric cancer syndrome, but mutations have been also identified in familial invasive lobular BrCa patients without hereditary diffuse gastric cancer [15,43]. Here, only neutral variants were identified, and all of them have been reported earlier [see Supplementary Table S1 in Additional file 1]. No clear results were found that any of the identified genetic variants in *BRIP1*, *RAD50*, or *CDH1 *would increase the BrCa/OvCa risk in the analyzed high-risk Finnish HBOC individuals.

## Conclusions

In this study, 13.4% of the analyzed, high-risk *BRCA1/2*-founder mutation-negative HBOC cases can be explained by previously reported mutations in BrCa susceptibility genes. *CHEK2 *mutations, c.470T > C and c.1100delC, make a considerable contribution (12.2%) to these high-risk individuals but further segregation analysis are needed to evaluate the clinical significance of these mutations before applying them in clinical use. Novel variant findings warrant additional studies with special interest in the novel missense variant, *BRCA2 *c.72A > T (Leu24Phe), which was predicted to bear untolerated mutations and to destabilize the protein. The complex nature of HBOC addresses the need for genome-wide approaches to further study these individuals and to create new tools for genetic counseling. This study also confirmed that our current genetic testing protocol for the 28 Finnish *BRCA1/2*-founder mutations and PTT of the largest exons is sensitive enough for clinical use in the majority of Finnish HBC/HBOC individuals.

## Abbreviations

*BRCA1*: breast cancer 1 gene; *BRCA2*: breast cancer 2 gene; BrCa: breast cancer; *BRIP1*: BRCA1-interacting protein 1 gene; *CDH1*: cadherin-1 gene; *CHEK2*: checkpoint kinase 2 gene; CI: confidence interval; *FGFR2*: fibroblast growth factor receptor 2 gene; GWAs: genome-wide association studies; HBC: hereditary breast cancer; HBOC: high-risk hereditary breast and/or ovarian cancer; HRM: high resolution melt; miRNA: microRNA; MLPA: multiplex ligation-dependent probe amplification; OR: odds ratio; OvCa: ovarian cancer; PALB2: Partner and localizer of BRCA2; PCR: polymerase chain reaction; PTT: protein truncation test; *RAD50*: human homolog of S. cerevisiae RAD50 gene; SNP: single nucleotide polymorphism.

## Competing interests

The authors declare that they have no competing interests.

## Authors' contributions

KMK participated patient collection, carried out the sequencing, MLPA and statistical analysis and drafted the manuscript. AB carried out and interpreted the HRM analysis of the *CHEK2 *gene and helped to draft the methods section of the manuscript. MV performed and interpreted protein prediction analysis *in silico *and helped to draft the manuscript. JS and S-L S participated in the study design and coordination, and helped to draft the manuscript. S-L S also participated in patient collection and was responsible for genetic counseling of patients. All authors read and approved the final manuscript.

## Supplementary Material

Additional file 1 **Supplementary Table S1. All of the identified 54 sequence alterations**. Supplementary Table S1 include detailed information about all of the identified sequence alterations. Click here for file

Additional file 2 **Supplementary Table S2. Identified breast cancer associated variants in affected 71 individuals**. Supplementary Table S2 includes re-calculated frequencies for *BRCA1 *c.5095C > T, *CHEK2 *c.470T > C, and *CHEK2 *c.1100delC variants in affected 71 index individuals (11 unaffected index individuals excluded). Click here for file
