# Supplementary material for: Screening for BRCA1, BRCA2, CHEK2, PALB2, BRIP1, RAD50, and CDH1 mutations in high-risk Finnish BRCA1/2-founder mutation-negative breast and/or ovarian cancer individuals
Source: Breast Cancer Res. 2011 Feb 28;13(1):R20. doi: 10.1186/bcr2832 (PMC3109589; doi:10.1186/bcr2832)
Supplement: Additional file 1 — Supplementary Table S1. All of the identified 54 sequence alterations. Supplementary Table S1 include detailed information about all of the identified sequence alterations. [file bcr2832-S1.PDF]

Supplementary Table 1. All of the identified 54 sequence alterations.

Gene/				Carrier Frequency				
Exon/Intron/UTR	Nucleotide change[a]	Effect on protein	rs Number[b]	Individuals	Controls	P-values	OR;95%CI	Status
BRCA1								
Exon 9	591C>T	Cys197Cys	rs1799965	0.012 (1/82)	na	-	-	Reported[c, d]
				TT 0 (0/82)				
				CT 0.012 (1/82)				
				CC 0.988 (81/82)				
Exon 13	4308T>C	Ser1436Ser	rs1060915	0.634 (52/82)	na	-	-	Reported[c, d]
				CC 0.122 (10/82)				
				TC 0.512 (42/82)				
				TT 0.366 (30/82)				
Intron 13	4357+117G>A	-	rs3737559	0.207 (17/82)	na	-	-	Reported[c]
				AA 0 (0/82)				
				GA 0.207 (17/82)				
				GG 0.793 (65/82)				
Intron 14	4485-63G>C	-	rs8176212	0.610 (50/82)	na	-	-	Reported[c]
				CC 0.122 (10/82)				
				GC 0.488 (40/82)				
				GG 0.390 (32/82)				
Exon 16	4837A>G	Ser1613Gly	rs1799966	0.634 (52/82)	na	-	-	Reported[c, d]
				GG 0.122 (10/82)				
				AG 0.512 (42/82)				
				AA 0.366 (30/82)				
Exon 16	4883T>C	Met1628Thr	rs4986854	0.049 (4/82)	0.016 (6/367)	0.090	3.09; 0.85-11.19	Reported[c, d]
				CC 0 (0/82)	CC 0 (0/367)			
				TC 0.049 (4/82)	TC 0.016 (6/367)			
				TT 0.951 (78/82)	TT 0.984 (361/367)			
Exon 18	5095C>T	Arg1699Trp	rs55770810	0.012 (1/82)	na	-	-	Reported[c, d]
				TT 0 (0/82)				
				CT 0.012 (1/82)				

				CC 0.988 (81/82)				
BRCA2								
5'UTR	-26G>A	-	rs1799943	0.341 (28/82)	na	-	-	Reported[c]
				AA 0.037 (3/82)				
				GA 0.304 (25/82)				
				GG 0.659 (54/82)				
Intron 2	68-80insT[g]	-	-	0.012 (1/82)	na	-	-	Novel
Exon 3	72A>T	Leu24Phe	-	0.012 (1/82)	0 (0/380)	0.177	na	Novel
				TT 0 (0/82)	TT 0 (0/380)			
				AT 0.012 (1/82)	AT 0 (0/380)			
				AA 0.988 (81/82)	AA 0 (0/380)			
Intron 8	681+56C>T	-	rs2126042	0.451 (37/82)	na	-	-	Reported[c, d]
				TT 0.024 (2/82)				
				CT 0.427 (35/82)				
				CC 0.549 (45/82)				
Intron 9	793+34T>G	-	-	0.012 (1/82)	na	-	-	Novel
				GG 0 (0/82)				
				TG 0.012 (1/82)				
				TT 0.988 (81/82)				
Exon 14	7242A>G	Ser2414Ser	rs1799955	0.427 (35/82)	na	-	-	Reported[c, d]
				GG 0.049 (4/82)				
				AG 0.378 (31/82)				
				AA 0.573 (47/82)				
Exon 18	8182G>A	Val2728Ile	rs28897749	0.012 (1/82)	0.003 (1/378)	0.325	4.65; 0.29-75.19	Reported[c, d]
				AA 0 (0/82)	AA 0 (0/378)			
				GA 0.012 (1/82)	GA 0.003 (1/378)			
				GG 0.988 (81/82)	GG 0.997 (377/378)			
Exon 27	9976A>T	Lys3326Stop	rs11571833	0.012 (1/82)	0.029 (11/378)	0.702	0.41; 0.05-3.24	Reported[c, d]
				TT 0 (0/82)	TT 0 (0/378)			
				AT 0.012 (1/82)	AT 0.029 (11/378)			
				AA 0.988 (81/82)	AA 0.971 (367/378)			

Exon 27	10234A>G	Ile3412Val	rs1801426	0.012 (1/82)	0.021 (8/379)	1.000	0.57; 0.07-4.64	Reported[c, d]
				GG 0 (0/82)	GG 0 (0/379)			
				AG 0.012 (1/82)	AG 0.021 (8/379)			
				AA 0.988 (81/82)	AA 0.979 (371/379)			
CHEK2								
Intron 1	319+43_319+44insA[g]	-	rs17879991	0.293 (24/82)	0.301 (112/372)	1.000	0.96; 0.57-1.62	Reported[c]
Intron 2	444+85T>A	-	-	0.012 (1/82)	0.005 (2/364)	0.457	2.23; 0.20-24.94	Novel
				AA 0 (0/82)	AA 0 (0/364)			
				TA 0.012 (1/82)	TA 0.005 (2/364)			
				TT 0.988 (81/82)	TT 0.995 (362/364)			
Exon 3	470T>C	Ile157Thr	-	0.098 (8/81)	0.055 (21/381)	0.203	1.88; 0.80-4.41	Reported[e]
				CC 0 (0/81)	CC 0 (0/381)			
				TC 0.099 (8/81)	TC 0.055 (21/381)			
				TT 0.901 (73/81)	TT 0.945 (360/381)			
Intron 5	792+39C>T	-	-	0.012 (1/82)	0.021 (8/375)	1.000	0.57; 0.07-4.60	Novel
				TT 0 (0/82)	TT 0 (0/375)			
				CT 0.012 (1/82)	CT 0.021 (8/375)			
				CC 0.988 (81/82)	CC 0.979 (367/375)			
Exon 10	1100delC[g]	Fs, stop at codon 381	-	0.037 (3/82)	0.016 (6/380)	0.203	2.37; 0.58-9.67	Reported[e]
Exon 11	1290T>C	His430His	-	0.951 (77/81)	0.974 (372/382)	0.281	0.52; 0.16-1.69	Novel
				CC 0.025 (2/81)	CC 0.024 (9/382)			
				TC 0.926 (75/81)	TC 0.950 (363/382)			
				TT 0.049 (4/81)	TT 0.026 (10/382)			
Exon 11	1314T>C	Asp438Asp	-	0.951 (77/81)	0.974 (372/382)	0.281	0.52; 0.16-1.69	Novel
				CC 0.025 (2/81)	CC 0.024 (9/382)			
				TC 0.926 (75/81)	TC 0.950 (363/382)			
				TT 0.049 (4/81)	TT 0.026 (10/382)			
Exon 11	1363G>A	Val455Ile	-	0.975 (79/81)	0.976 (373/382)	1.000	0.95; 0.20-4.50	Novel
				AA 0 (0/81)	AA 0 (0/382)			
				GA 0.975 (79/81)	GA 0.976 (373/382)			
				GG 0.025 (2/81)	GG 0.024 (9/382)			

PALB2								
5'UTR	-47G>A	-	rs8053188	0.049 (4/82)	na	-	-	Reported[c]
				AA 0 (0/82)				
				GA 0.049 (4/82)				
				GG 0.951 (78/82)				
Exon 4	814G>A	Glu272Lys	-	0.012 (1/82)	0 (0/372)	0.181	na	Novel
				AA 0 (0/82)	AA 0 (0/372)			
				GA 0.012 (1/82)	GA 0 (0/372)			
				GG 0.988 (81/82)	GG 0 (0/372)			
Exon 4	1000T>G	Tyr334Asp	-	0.012 (1/82)	0.011 (4/380)	1.000	1.16; 0.13-10.52	Novel
				GG 0 (0/82)	GG 0 (0/380)			
				TG 0.012 (1/82)	TG 0.011 (4/380)			
				TT 0.988 (81/82)	TT 0.989 (376/380)			
Exon 4	1010T>C	Leu337Ser	rs45494092	0.073 (6/82)	na	-	-	Reported[c]
				CC 0 (0/82)				
				TC 0.073 (6/82)				
				TT 0.927 (76/82)				
Exon 4	1676A>G	Gln559Arg	rs152451	0.122 (10/82)	0.173 (64/371)	0.323	0.67; 0.33-1.36	Reported[c]
				GG 0.012 (1/82)	GG 0.011 (4/371)			
				AG 0.110 (9/82)	AG 0.162 (60/371)			
				AA 0.878 (72/82)	AA 0.827 (307/371)			
Exon 5	2205A>G	Pro735Pro	-	0.012 (1/82)	na	-	-	Novel
				GG 0 (0/82)				
				AG 0.012 (1/82)				
				AA 0.988 (81/82)				
Intron 6	2586+58C>T	-	rs249954	0.439 (36/82)	na	-	-	Reported[c]
				TT 0.049 (4/82)				
				CT 0.390 (32/82)				
				CC 0.561 (46/82)				
Exon 8	2794G>A	Val932Met	rs45624036	0.037 (3/82)	na	-	-	Reported[c]
				AA 0 (0/82)				
				GA 0.037 (3/82)				

				GG 0.963 (79/82)				
Exon 9	2993G>A	Gly998Glu	rs45551636	0.012 (1/82)	0.038 (14/372)	0.491	0.32; 0.04-2.44	Reported[c]
				AA 0 (0/82)	AA 0 (0/372)			
				GA 0.012 (1/82)	GA 0.038 (14/372)			
				GG 0.988 (81/82)	GG 0.962 (358/372)			
BRIP1								
Exon 6	584T>C	Leu195Pro	rs4988347	0.024 (2/82)	na	-	-	Reported[c]
				CC 0 (0/82)				
				TC 0.024 (2/82)				
				TT 0.976 (80/82)				
Exon 19	2755C>T	Pro919Ser	rs4986764	0.390 (32/82)	na	-	-	Reported[c]
				TT 0.207 (17/82)				
				CT 0.183 (15/82)				
				CC 0.610 (50/82)				
Exon 19	2637G>A	Glu879Glu	rs4986765	0.683 (56/82)	na	-	-	Reported[c]
				AA 0.415 (34/82)				
				GA 0.268 (22/82)				
				GG 0.317 (26/82)				
Exon 20	3411C>T	Tyr1137Tyr	rs4986763	0.793 (65/82)	na	-	-	Reported[c]
				TT 0.305 (25/82)				
				CT 0.488 (40/82)				
				CC 0.207 (17/82)				
RAD50								
Intron 4	551+19G>A	-	rs17166050	0.439 (36/82)	na	-	-	Reported[c]
				AA 0.098 (8/82)				
				GA 0.341 (28/82)				
				GG 0.561 (46/82)				
Exon 10	1544A>G	Asp515Gly	-	0.012 (1/82)	0.010 (4/384)	1.000	1.17; 0.13-10.63	Novel
				GG 0 (0/82)	GG 0 (0/384)			
				AG 0.012 (1/82)	AG 0.010 (4/384)			

				AA 0.988 (81/82)	AA 0.990 (380/384)			
Intron 14	2398-32A>G	-	-	0.012 (1/82)	na	-	-	Novel
				GG 0 (0/82)				
				AG 0.012 (1/82)				
				AA 0.988 (81/82)				
Intron 16	2719-31A>G	-	rs104895047	0.012 (1/82)	na	-	-	Reported[c]
				GG 0 (0/82)				
				AG 0.012 (1/82)				
				AA 0.988 (81/82)				
Intron 20	3164+49G>C	-	-	0.037 (3/82)	na	-	-	Reported[f]
				CC 0 (0/82)				
				GC 0.037 (3/82)				
				GG 0.963 (79/82)				
Intron 22	3475+24A>G	-	-	0.110 (9/82)	na	-	-	Reported[f]
				GG 0 (0/82)				
				AG 0.110 (9/82)				
				AA 0.890 (73/82)				
Intron 22	3475+33C>G	-	-	0.012 (1/82)	na	-	-	Novel
				GG 0 (0/82)				
				CG 0.012 (1/82)				
				CC 0.988 (81/82)				
CDH1								
5'UTR	-71C>G	-	rs34033771	0.085 (7/82)	na	-	-	Reported[c]
				GG 0 (0/82)				
				CG 0.085 (7/82)				
				CC 0.915 (75/82)				
Intron 1	48+6C>T	-	rs3743674	0.951 (78/82)	na	-	-	Reported[c]
				TT 0.732 (60/82				
				CT 0.219 (18/82)				
				CC 0.049 (4/82)				
Intron 4	531+10G>C	-	rs33963999	0.098 (8/82)	na	-	-	Reported[c]

				CC 0 (0/82)				
				GC 0.098 (8/82)				
				GG 0.902 (74/82)				
Intron 11	1711+47G>A	-	rs35667437	0.012 (1/82)	na	-	-	Reported[c]
				AA 0 (0/82)				
				GA 0.012 (1/82)				
				GG 0.988 (81/82)				
Exon 12	1896C>T	His632His	rs33969373	0.012 (1/82)	na	-	-	Reported[c]
				TT 0 (0/82)				
				CT 0.012 (1/82)				
				CC 0.988 (81/82)				
Intron 12	1937-13T>C	-	rs2276330	0.171 (14/82)	na	-	-	Reported[c]
				CC 0 (0/82)				
				TC 0.171 (14/82)				
				TT 0.829 (68/82)				
Exon 13	2076T>C	Ala692Ala	rs1801552	0.866 (71/82)	na	-	-	Reported[c]
				CC 0.354 (29/82)				
				TC 0.512 (42/82)				
				TT 0.134 (11/82)				
Intron 13	2164+17_2164+18insA[g]	-	rs34939176	0.024 (2/82)	na	-	-	Reported[c]
Intron 15	2439+52A>G	-	rs33965115	0.073 (6/82)	na	-	-	Reported[c]
				GG 0 (0/82)				
				AG 0.073 (6/82)				
				AA 0.927 (76/82)				
Exon 16	2634C>T	Gly878Gly	rs2229044	0.012 (1/82)	na	-	-	Reported[c]
				TT 0 (0/82)				
				CT 0.012 (1/82)				
				CC 0.988 (81/82)				

CI: confidence interval, Fs: frameshift, na: not analyzed, OR:odds ratio. [a]The reference nucleotide sequencies were obtained from the UCSC Genome Browser [44] and the accession numbers were following: *BRCA1*: [UCSC Genome Browser:NM_007295.2], *BRCA2*: [UCSC Genome Browser:NM_000059.3], *CHEK2*: [UCSC Genome Browser:NM_007194.3], *PALB2*: [UCSC Genome Browser:NM_024675.3], *BRIP1*: [UCSC Genome Browser:NM_032043.1], *RAD50*: [UCSC Genome Browser:NM_005732.3], and *CDH1*: [UCSC Genome Browser:NM_004360.3]. The accession numbers for the protein sequencies obtained from the Swiss-Prot Protein knowledgebase [45] were following: *BRCA1*: [Swiss-Prot:P38398], *BRCA2*: [Swiss-Prot:P51587], *CHEK2*: [Swiss-Prot:O96017], *PALB2*: [Swiss-Prot:Q86YC2**]**, *BRIP1*: [Swiss-Prot:Q9BX63**],** *RAD50*:

[Swiss-Prot:Q92878**],** and *CDH1*: [Swiss-Prot:P12830]. [b]The RefSNP number, obtained from the NCBI Single Nucleotide Polymorphism database (dbSNP) [46]. [c]The NCBI dbSNP [46]. [d]The Breast Cancer Information Core database [47]. [e]Reported in the Finnish population by Vahteristo et al.[11]. [f]Reported by Tommiska et al.[19] . [g]Heterozygous deletion or insertion. Numbering of exons and introns may vary compared to database.
