# Supplementary material for: Screening for BRCA1, BRCA2, CHEK2, PALB2, BRIP1, RAD50, and CDH1 mutations in high-risk Finnish BRCA1/2-founder mutation-negative breast and/or ovarian cancer individuals
Source: Breast Cancer Res. 2011 Feb 28;13(1):R20. doi: 10.1186/bcr2832 (PMC3109589; doi:10.1186/bcr2832)
Supplement: Additional file 2 — Supplementary Table S2. Identified breast cancer associated variants in affected 71 individuals. Supplementary Table S2 includes re-calculated frequencies for BRCA1 c.5095C > T, CHEK2 c.470T > C, and CHEK2 c.1100delC variants in affected 71 index individuals (11 unaffected index individuals excluded). [file bcr2832-S2.PDF]

Supplementary Table 2. Identified breast cancer associated variants in affected 71 individuals.

Gene/ Exon/Intron/UTR	Nucleotide change[a]	Effect on protein	rs Number[b]	Carrier Frequency Individuals	Controls	P-values	OR;95%CI	Status
BRCA1								
Exon 18	5095C>T	Arg1699Trp	rs55770810	**0.014 (1/71)**	na	-	-	Reported[c, d]
				TT 0 (0/71)				
				CT 0.014 (1/71)				
				CC 0.986 (70/71)				
CHEK2								
Exon 3	470T>C	Ile157Thr	-	**0.071 (5/70)**	0.055 (21/381)	**0.578**	**1.32; 0.48-3.62**	Reported[e]
				CC 0 (0/70)	CC 0 (0/381)			
				TC 0.071 (5/70)	TC 0.055 (21/381)			
				TT 0.929 (65/70)	TT 0.945 (360/381)			
Exon 10	1100delC[f]	Fs, stop at codon 381	-	**0.042(3/71)**	0.016 (6/380)	**0.155**	**2.75; 0.67-11.26**	Reported[e]

CI: confidence interval, Fs: frameshift, na: not analyzed, OR:odds ratio. [a]The reference sequencies were obtained from the UCSC Genome Browser [44] and the accession numbers were following: *BRCA1*: [UCSC Genome Browser:NM_007295.2] and *CHEK2*: [UCSC Genome Browser:NM_007194.3]. The accession numbers for the protein sequencies obtained from the Swiss-Prot Protein knowledgebase [45] were following: *BRCA1*: [Swiss-Prot:P38398] and *CHEK2*: [Swiss-Prot:O96017], [b]The RefSNP number, obtained from the NCBI Single Nucleotide Polymorphism database (dbSNP) [46]. [c]The NCBI dbSNP [46]. [d]The Breast Cancer Information Core database [47]. [e]Reported in the Finnish population by Vahteristo et al.[11]. [f]Heterozygous deletion or insertion.
